# Transcriptional expression study in the central nervous system of rats: what gene should be used as internal control?

**DOI:** 10.1590/S1679-45082014AO3042

**Published:** 2014

**Authors:** Ana Carolina de Moura, Virgínia Meneghini Lazzari, Grasiela Agnes, Silvana Almeida, Márcia Giovenardi, Ana Beatriz Gorini da Veiga

**Affiliations:** 1 Universidade Federal de Ciências da Saúde de Porto Alegre, Porto Alegre, RS, Brazil.

**Keywords:** Rats, Wistar, Brain, Gene expression, Real-time polymerase chain reaction, Genes, essential

## Abstract

**Objective:**

A growing number of published articles report the expression of specific genes with different behavior patterns in rats. The levels of messenger ribonucleic acid transcripts are usually analyzed by reverse transcription followed by polymerase chain reaction and quantified after normalization with an internal control or reference gene (housekeeping gene). Nevertheless, housekeeping genes exhibit different expression in the central nervous system, depending on the physiological conditions and the area of the brain to be studied. The choice of a good internal control gene is essential for obtaining reliable results. This study evaluated the expression of three housekeeping genes (beta-actin, cyclophilin A, and ubiquitin C) in different areas of the central nervous system in rats (olfactory bulb, hippocampus, striatum, and prefrontal cortex).

**Methods:**

Wistar rats (virgin females, n=6) during the diestrum period were used. Total ribonucleic acid was extracted from each region of the brain; the complementary deoxyribonucleic acid was synthesized by reverse transcription and amplified by real-time quantitative polymerase chain reaction using SYBR™ Green and primers specific for each one of the reference genes. The stability of the expression was determined using NormFinder.

**Results:**

Beta-actin was the most stable gene in the hippocampus and striatum, while cyclophilin A and ubiquitin C showed greater stability in the prefrontal cortex and the olfactory bulb, respectively.

**Conclusion:**

Based on our study, further studies of gene expression using rats as animal models should take into consideration these results when choosing a reliable internal control gene.

## INTRODUCTION

Structures in the central nervous system (CNS) such as olfactory bulb (OB), hippocampus (HP), striatum (ST), prefrontal cortex (PFC), posterodorsal medial amygdala (MePD), and medial preoptic area (MPOA) are responsible for the appearance and maintenance of different behaviors.^(1, 2)^ Understanding the molecular mechanisms involved in the regulation of signaling pathways in the CNS has been the basis of many studies, which aimed to address how a pattern of behavior is controlled by the expression of a candidate gene or group of genes.^(3)-5)^ Therefore, gene expression assays have been increasingly employed in behavioral studies using animal models, based on analysis of specific transcripts in the CNS and their association with different patterns of behavior.

Levels of expressed genes within a cell can be altered by a variety of conditions, such as the cell cycle phase or upon exposure to drugs, hormones, cytokines or other stimuli. Therefore, gene expression analysis requires precise and reproducible measurements of specific messenger ribonucleic acid (mRNA) sequences. The most common method used to quantify mRNA is the amplification of individual RNA molecules by combining reverse transcription and real-time polymerase chain reaction (RT-PCR),^(6)^ which enables a sensitive and accurate quantification of mRNA expression levels. Nevertheless, the selection of an appropriate normalization strategy is necessary, in order to exclude possible experimental errors, thus providing more trustworthy data. Most gene expression experiments require ribonucleic acid (RNA) isolation and processing, and the final amount of RNA may vary among samples. Performing RT-PCR analysis requires controlled parameters to obtain reliable quantitative expression measures. These include variations in initial sample amount, RNA recovery, RNA integrity, efficiency of complementary deoxyribonucleic acid (cDNA) synthesis, and differences in the overall transcriptional activity of the tissues or cells analyzed.^(7, 8)^

The most frequently applied approach for normalization in studies of gene expression is the use of an internal control or an endogenous reference gene, often referred to as a housekeeping gene (HKG). HKG encode proteins that provide basic, essential functions that all cells need to survive. They are supposed to have stable expression levels in different cell types and tissues, across developmental stages and even under various conditions. However, several studies have shown that transcript levels of traditionally used HKG may vary considerably in specific cells under different experimental conditions.^(7)-10)^

In recent years, it has become clear that no single gene is constitutively expressed in all cell types and under all experimental conditions, implying that the expression stability of the intended control gene has to be verified before each particular experimental model.^(7)^ For a gene to be used as an internal control, several criteria should be fulfilled: mRNA must be consistently expressed at the same level in all samples under investigation, regardless of tissue type, disease state, medication, or experimental conditions; expression levels must be comparable to that of the target; and the amplification of the reference gene should be RNA-specific. The importance of choosing a reliable internal control is underlined by the fact that the use of an unstable reference gene for normalization may obscure real changes or produce artificial modifications in gene expression. Therefore, the validation of an HKG for each experimental situation is a crucial requirement for the acquisition of meaningful biological data.^(8, 11)^ For more generalized studies, the use of the geometric mean level of expression from several genes for normalization, as well as the use of three to five different control genes, depending on the tissue being studied, are recommended.^(12)^

Until now, a systematic study comparing the suitability of candidate reference genes in the rat central nervous system has not been performed. The results presented in this study can be helpful in further analyses of gene expression in rats’ brain.

## OBJECTIVE

The aim of the present study was to select a sample set to analyze distinct brain regions and evaluate the most stable housekeeping gene in areas of the central nervous system related with various social behaviors.

## METHODS

### Animals

Virgin female Wistar rats (n=6) with at least 3 consecutive regular cycles, in the diestrus period, approximately 90 days old, from the animal house of the *Universidade Federal de Ciências da Saúde de Porto Alegre* (UFCSPA), in Porto Alegre (RS), Brazil, were used. They were housed in groups of three in Plexiglas™ cages (46cm × 17cm × 31cm). Food and water were provided *ad libitum*. Animals were housed under controlled temperature (21±1°C) and light (12:12 light-dark cycle with lights off at 5:00 pm) conditions.

All procedures were performed from 2009 to 2012, in conformity with the Brazilian Society of Neuroscience and Behavior Guidelines for the care and use of laboratory animals, and the protocols were approved by the Research Ethics Committee of UFCSPA (protocol 590/08).

### Reference gene selection and primer design

Candidate reference genes were selected from those most commonly used in the literature. Primers for cyclophilin A (CypA) were as published by Peinnequin et al.^(13)^ and Langnaese et al.^(8)^ The beta-actin (ActB) and ubiquitin C (UbC) primers were designed using Primer3 software, based on rat sequences in the GenBank database. The specificity of the primers was checked using the Basic Local Alignment Search Tool (BLAST) search against nucleotide collection (nr) of the National Center for Biotechnology Information (NCBI) database. All primers were from Invitrogen™ (São Paulo, Brazil). The sequences of primers used are listed on table 1.

**Table 1:** Housekeeping genes selected for this study, sequences of primers, and gene function

HKG	Primer F	Primer R	Function (Langnaese et al.)^(8)^
ActB	5’TATGCCAACACAGTGCTGTCTGG3’	5’TACTCCTGCTTGCTGATCCACAT3’	Cytoskeletal structural protein
CypA	5’TATCTGCACTGCCAAGACTGAGTG3’	5’CTTCTTGCTGGTCTTGCCATTCC3’	Accelerate folding in oligopeptides
UbC	5’TTTCCATAGACAATGCAGATCTTT3’	5’AGGGTGGACTCCTTCTGGAT3’	Protein degradation

ActB: beta-actin; CypA: cyclophilin A UbC: ubiquitin C.

### Brain tissue samples

In order to determine the regularity of the estrous cycle, vaginal smears were taken from virgin female rats during 2 weeks before the beginning of the experiment. After the occurrence of three regular estrous cycles, experiments were performed on the morning of the diestrus phase. Rats were decapitated and their brains were quickly removed. The regions OB, HP, ST, and PFC were dissected from the left hemisphere. Soon after the samples were dissected, they were placed in tubes containing RNAlater™ (Life Technologies, São Paulo, Brazil) and stored at -80°C.

### Molecular analyses

Total RNA was extracted from samples using TRIzol™ (Invitrogen™, São Paulo, Brazil). Briefly, each brain structure was homogenized in the presence of TRIzol™, chloroform was added (1:5, v/v), and the aqueous phase was obtained after centrifugation (12,000×*g*, 15 minutes). RNA was precipitated with isopropanol for 15 minutes at room temperature, followed by centrifugation at 12,000×*g* for 10 minutes. Pellets were resuspended in 0.1% diethylpyrocarbonate (DEPC)-treated water, and RNA was quantified by spectrophotometry. The concentration of total RNA was determined by measuring the optical density at 260nm and the RNA purity was assessed based on the 260nm/280nm ratio and agarose gel electrophoresis.^(8)^

Semi-quantitative RT-PCR has been well accepted and widely used as a valid method for analysis of gene expression in brain areas, as described elsewhere.^(14)-16)^ For reverse transcription, 1μg of total RNA was used as template to synthesize cDNA. RNA was first incubated with 1μL of oligo(dT) (0.5μg/μL, Invitrogen™, São Paulo, Brazil), 1μL of deoxyribonucleotide triphosphate (dNTPs) (10mM) and DEPC-water to a final volume of 12μL for 5 minutes at 65°C and then for 1 minute in ice. The following reagents were then added to reach a final volume of 19μL:4μL of RT-PCR buffer (50mM TrisHCl, pH 8.3, 75mM KCl, 3mM MgCl_2_) and 2μL of DTT™ (0.1M). After 2 minutes of incubation at 37°C, 1μL of the enzyme reverse transcriptase encoded by *moloney murine leukemia virus reverse transcriptase* (M-MLV-RT; 200U/μL, Invitrogen™, São Paulo, Brazil) was added and cDNA synthesis was performed at 50°C for 1 hour; the reaction was inactivated by incubation at 70°C for 15 minutes.

Amplification of reference genes was carried out using 7.5μL of SYBR™ green polymerase chain reaction (PCR) master mix (Applied Biosystems™, São Paulo, Brazil), 0.5μL of forward and reverse primers (0.33µM each), 100ng of cDNA and nuclease-free water, in a total volume of 15μL. Reactions were performed in an optical 96-well plate, using a StepOnePlus™ thermocycler (Applied Biosystems™, Foster City, CA, USA). After an initial denaturation step at 95°C for 10 minutes, amplification was performed in 50 cycles of denaturation at 95°C for 30 seconds, annealing at 60°C for 40 seconds and extension at 72°C for 40 seconds. Amplification was followed by a melting curve analysis to confirm PCR product specificity. No signals were detected in no-template controls. The experimental threshold cycle (Ct) was calculated using the algorithm enhancements provided by the equipment. All samples were run in duplicate and the mean value of each duplicate was used for all further calculations.^(8, 10, 17)^

### Determination of reference gene expression stability

The publicly available software tool, NormFinder (a Visual Basic application for Microsoft Excel), was used to analyze gene expression stability. It ranks HKG according to their expression stability using a model-based approach. The program estimates both the intra-and the inter-group expression variation and calculates candidate gene stability values.^(7, 8)^

Reporting the data obtained from the raw Ct values falsely represents the variation and error, so it should be avoided. This way, the expression of each HKG was calculated from the formula 2^-Ct^. This calculation transforms the logarithmic Ct data to a linear value. The 2^-Ct^ form more accurately describes the individual variation among replicate reactions.^(6, 18)^ Thus, NormFinder requires the transformation of Ct values to linear scale expression quantities, so the average Ct values of the duplicates were therefore exported into Microsoft Excel, and changed to the 2^-Ct^ form, then the calculated quantities were entered into NormFinder,^(19)^ where the candidate most stably expressed in the sample set investigated is the top ranked gene, which has the smallest stability value.^(20)^

## RESULTS

**Figure 1:**
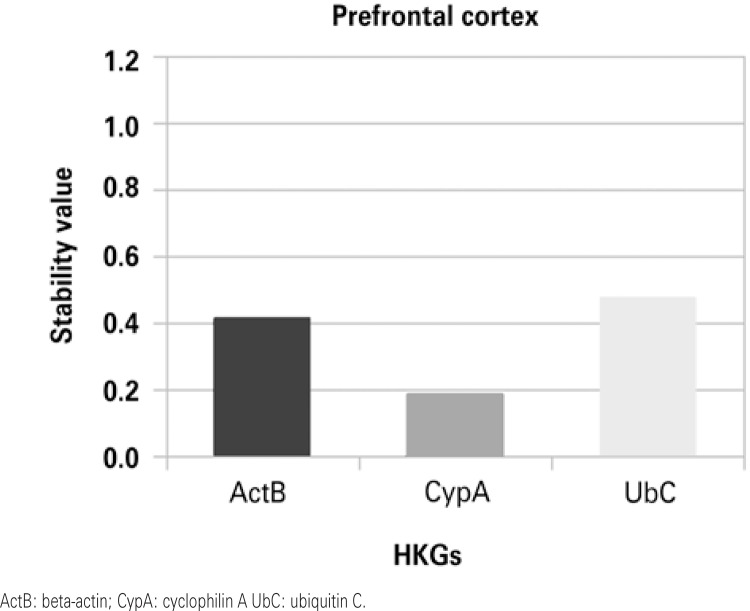
Housekeeping genes stability in the prefrontal cortex

**Figure 2:**
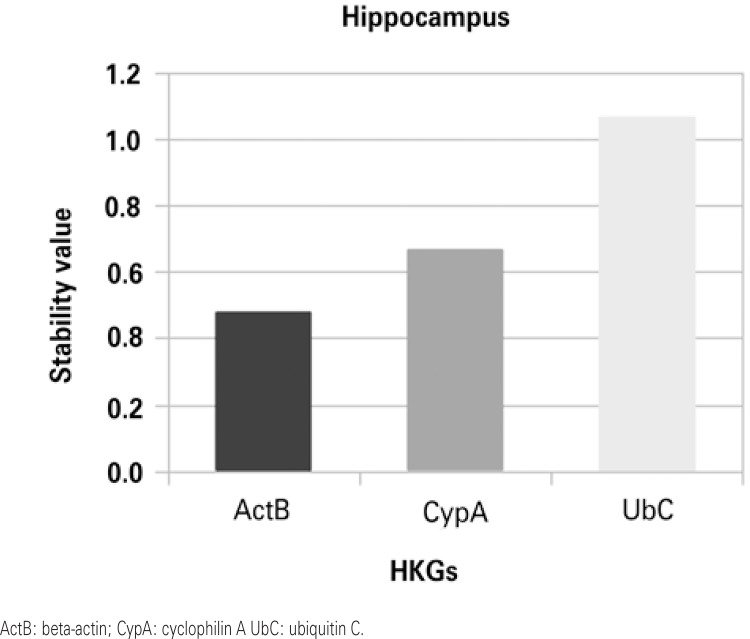
Housekeeping genes stability in the hippocampus

**Figure 3:**
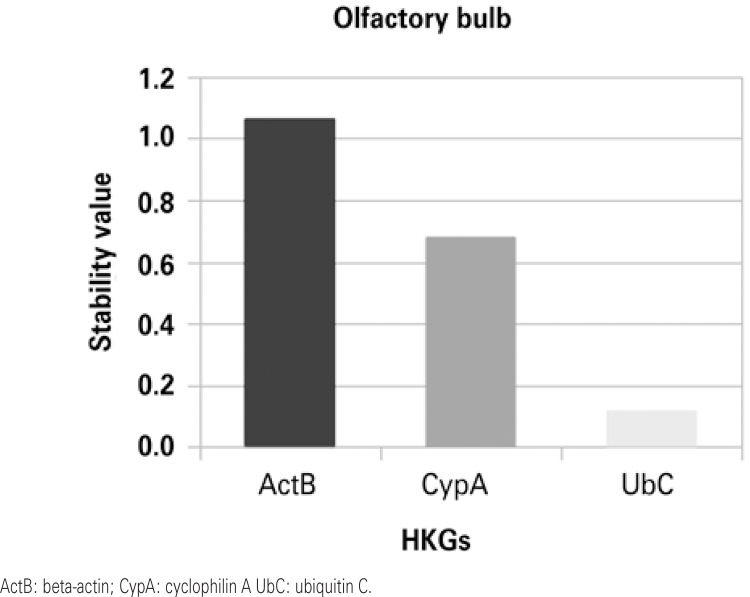
Housekeeping gene stability in the olfactory bulb

**Figure 4:**
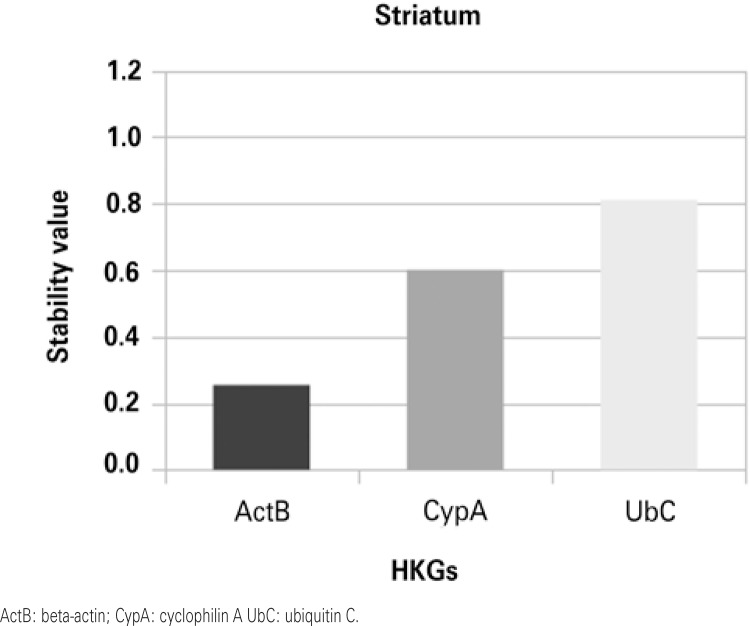
Housekeeping gene stability in the striatum

Stability values were obtained by ranking candidate normalization genes with NormFinder. Figures 1 to 4 show that in the HP and in the ST the most stable HKG was ActB. On the other hand, Cyp A and UbC were more stable in the PFC and in the OB, respectively.

## DISCUSSION

In some gene expression studies in the CNS, ActB was shown to be as stable as glyceraldehyde-3-phosphate dehydrogenase (GAPDH), but its stability depends on the tissue and conditions used in the analyses.^(21, 22)^ Chen et al.^(23)^ found that other constitutive genes, such as eukaryotic translation elongation factor (EF) and GAPDH, are more stable than ActB in some areas of the brain such as the auditory cortex and the cochlea of rats, and thus should be considered as reference genes in quantitative gene expression analyses in these regions. Yet, many studies based on RT-PCR to evaluate gene expression in different brain regions – such as PFC, ST, HP, and cerebellum – use ActB as reference gene.^(14)-16, 24)^

Our results show that ActB is the most stable HKG in the HP of our sample set. In a study by Langnaese et al.,^(8)^ ActB and CypA were shown to be the most stable genes in the HP of their analysis. These two HKG were also used as control genes in a study by Nishida et al.,^(25)^ because their expression levels are considered stable in this brain area. On the other hand, Honkaniemi et al.^(26)^ used only CypA in an ischemia model that analyzes gene expression in the HP.

In ST we also found ActB as the most stably expressed gene. However, Benn et al.^(9)^ identified that UbC is more stable than ActB in this area. Honkaniemi et al.^(26)^ examined the expression of CypA to compare the ischemic changes with general transcriptional levels in the ST.

CypA displayed the most stable expression in the PFC according to our analysis. Prior studies on gene expression levels made use mainly of one of the traditional HKG such as GAPDH, 18S, or CypA in the whole brain of adult Wistar rats.^(17)^ Honkaniemi et al.^(26)^ also examined the expression of CypA in the cortex of rats in an ischemic model. Furthermore, Benn et al.^(9)^ identified UbC as being stably expressed in the cortex, rather than ActB. But Yamada et al.^(27)^ used ActB as a reference for gene amplification, as well as Soria-Fregozo et al.,^(14)^ who chose ActB as an internal control for quantifying mRNA in the PFC.

We found that UbC is the most stable HKG in the OB. UbC has been used in several studies of gene expression, such as in the human brain^(24, 28)^ and rat liver.^(29)^ But Wong et al.^(30)^ analyzed the expression of ActB and CypA in the OB of rats.

Our study had some limitations. After RNA extraction with TRIzol**™**, samples were not incubated with DNAse, however the purity of the RNA was checked by agarose gel electrophoresis and no DNA was present in the samples. Another limitation was that the analyses were based on semi-quantitative RT-PCR. Even though more recent studies on gene expression are based on quantitative PCR, many studies have been based on conventional RT-PCR; as a matter of fact, this had been the most used molecular technique for gene expression analyses until recently, therefore its use does not impair our results or other previous findings.

## CONCLUSIONS

The variations on the stability of housekeeping genes in the different central nervous system areas probably occur due to the variability in the samples used in each study. Therefore, the validation of reference genes for each experimental situation is a crucial requirement for the acquisition of meaningful and reliable data for any sort of analysis. Once the housekeeping gene has been selected for each central nervous system area, further analysis relating the expression of target genes in animal models can be accomplished with the security of using a stable control gene.
