# Supplementary material for: Transcriptional expression study in the central nervous system of rats: what gene should be used as internal control?
Source: Einstein (Sao Paulo). 2014 Jul-Sep;12(3):336–41. doi: 10.1590/S1679-45082014AO3042 (PMC4872946; doi:10.1590/S1679-45082014AO3042)
Supplement: Translation

# Translation: Estudo de expressão transcricional no sistema nervoso central de ratos: qual gene deve ser usado como controle interno?

**Authors:** Ana Carolina de Moura, Virgínia Meneghini Lazzari, Grasiela Agnes, Silvana Almeida, Márcia Giovenardi, Ana Beatriz Gorini da Veiga

**Affiliations:** 1 Universidade Federal de Ciências da Saúde de Porto Alegre, Porto Alegre, RS, Brasil.

**Keywords:** Ratos Wistar, Encéfalo, Expressão gênica, Reação em cadeia da polimerase em tempo real, Genes essenciais

## Abstract

**Objetivo:**

Um número crescente de artigos publicados relaciona a expressão de genes específicos com diferentes padrões de comportamento em ratos. Os níveis de transcritos de ácido ribonucleico mensageiro são geralmente analisados por transcrição reversa, seguida de reação em cadeia da polimerase, e quantificados após a normalização com um controle interno ou gene de referência (gene *housekeeping*). No entanto, os genes *housekeeping* exibem expressão diferencial no sistema nervoso central, dependendo das condições fisiológicas e da área do cérebro a ser estudada. A escolha de um bom gene de controle interno é essencial para a obtenção de resultados confiáveis. Este estudo avaliou a expressão de três genes* housekeeping* (beta-actina, ciclofilina A e ubiquitina C) em diferentes áreas do sistema nervoso central de ratos (bulbo olfatório, hipocampo, estriado e córtex pré-frontal).

**Métodos:**

Foram usadas ratas Wistar (fêmeas virgens, n=6) durante o período de diestro. O ácido ribonucleico total foi extraído a partir de cada região do cérebro; o ácido desoxirribonucleico complementar foi sintetizado por transcrição reversa e amplificado por reação em cadeia da polimerase quantitativo em tempo real utilizando SYBR^® ^Green e* primers* específicos para cada um dos genes de referência. A estabilidade de expressão foi determinada utilizando NormFinder.

**Resultados:**

A beta-actina foi o gene mais estável no hipocampo e estriado, enquanto a ciclofilina A e a ubiquitina C apresentaram maior estabilidade no córtex pré-frontal e no bulbo olfatório, respectivamente.

**Conclusão:**

Com base em nosso trabalho, estudos posteriores de expressão gênica utilizando ratos como modelos animais devem levar estes resultados em conta na escolha de um gene de controle interno confiável.

## INTRODUÇÃO

Algumas estruturas no sistema nervoso central (SNC), como bulbo olfatório (OB), hipocampo (HP), estriado (ST), córtex pré-frontal (PFC), amígdala medial posterodorsal (MePD) e área pré-óptica medial (MPOA), são responsáveis pela aparência e pela manutenção de diferentes comportamentos.^(1, 2)^ A compreensão dos mecanismos moleculares envolvidos na regulação das vias de sinalização no SNC serviu de base para muitos estudos, que visaram analisar como um padrão de comportamento é controlado pela expressão de um gene candidato ou de grupo de genes.^(3)-5)^ Assim, ensaios sobre a expressão de genes são cada vez mais usados em estudos comportamentais com modelos animais, com base na análise de transcritos específicos no SNC e sua associação com diferentes padrões de comportamento.

Os níveis de expressão de um gene dentro de uma célula podem ser alterados por diversas condições, como a fase do ciclo celular ou a exposição a drogas, hormônios, citocinas ou outros estímulos. Logo, a análise da expressão de genes requer medidas precisas e reprodutíveis de sequências específicas de ácido ribonucleico mensageiro (mRNA). O método mais comum de quantificação de mRNA é a amplificação de moléculas individuais de RNA, pela combinação de transcrição reversa e pela reação da cadeia de polimerase em tempo real (RT-PCR),^(6)^ que permitem uma quantificação sensível e acurada dos níveis de expressão de mRNA. Mesmo assim, é necessário haver seleção de uma estratégia apropriada de normalização para excluir possíveis erros experimentais, fornecendo, desse modo, dados mais confiáveis. A maioria dos experimentos de expressão gênica requer isolamento e processamento do ácido ribonucleico (RNA), e a quantidade final de RNA pode variar entre as amostras. A realização de análises RT-PCR demanda parâmetros controlados para a obtenção de medidas quantitativas confiáveis de expressão. Estas incluem variações na quantidade da amostra inicial, recuperação de RNA, integridade de RNA, eficiência de síntese do ácido desoxirribonucleico complementar (cDNA) e diferenças na atividade geral de transcrição dos tecidos ou células analisados.^(7, 8)^

A abordagem usada mais frequentemente para normalização em estudos de expressão gênica é o emprego de um controle interno ou gene de referência endógeno, frequentemente chamado de gene *housekeeping* (HKG). Os HKG codificam proteínas que oferecem funções básicas, essenciais, das quais todas as células necessitam para sobreviver. Eles devem ter níveis de expressão estáveis em diferentes tipos de células e tecidos, por todos os estágios de desenvolvimento, e mesmo em várias condições. Entretanto, vários estudos mostraram que os níveis de transcrição de HKG usados tradicionalmente podem variar de forma considerável em células específicas em condições experimentais diferentes.^(7)-10)^

Nos últimos anos, ficou claro que nenhum gene isolado é expresso constitutivamente em todos os tipos de células e sob todas as condições experimentais, sugerindo que a estabilidade de expressão do gene de controle considerado tem de ser verificada antes de cada modelo experimental particular.^(7)^ Para que um gene seja usado como controle interno, diversos critérios devem ser satisfeitos: o mRNA tem de ser expresso de modo consistente no mesmo nível em todas as amostras sob investigação, independentemente do tipo de tecido, estado de doença, medicação ou condição experimental; os níveis de expressão têm de ser comparáveis aos do alvo; e a amplificação do dado gene de referência deve ser específica para o RNA. A importância da escolha de um controle interno confiável é salientada pelo fato de que o uso de um gene de referência instável para normalização pode obscurecer as reais alterações ou produzir modificações artificiais na expressão do gene. Assim, a validação de um HKG para cada situação experimental é um requerimento crucial para a aquisição de dados biológicos significantes.^(8, 11)^ Para estudos mais generalizados, são recomendados o uso do nível geométrico médio de expressão de vários genes para normalização e também o uso de três a cinco genes de controle diferentes, dependendo do tecido a ser estudado.^(12)^

Ainda não foi realizado um estudo sistemático que comparasse a capacidade de adequação de genes de referência candidatos no sistema nervoso central de ratos. Os resultados apresentados neste estudo podem ser úteis em análises posteriores de expressão gênica no cérebro de ratos.

## OBJETIVO

O objetivo do presente estudo foi selecionar uma amostra para analisar regiões distintas do cérebro e avaliar as áreas mais estáveis do gene *housekeeping* do sistema nervoso central, relacionadas a vários comportamentos sociais.

## MÉTODOS

### Animais

Foram usadas ratas Wistar virgens (n=6) com pelo menos 3 ciclos regulares consecutivos, no período diestro, com aproximadamente 90 dias de idade, do biotério da Universidade Federal de Ciências da Saúde de Porto Alegre (UFCSPA), em Porto Alegre (RS). Elas foram abrigadas em grupos de três em gaiolas de Plexiglas^® ^(46cm × 17cm × 31cm). Alimento e água foram oferecidos à vontade. Os animais foram abrigados em condições controladas de temperatura (21±1°C) e de luz (ciclos claro-escuro 12:12, com as luzes apagadas às 17h).

Todos os procedimentos foram realizados de 2009 a 2012, em conformidade com as Diretrizes da Sociedade Brasileira de Neurociências e Comportamento para o cuidado e uso de animais de laboratório, e os protocolos foram aprovados pelo Comitê de Ética em Pesquisa da UFCSPA (protocolo 590/08).

### Seleção de gene de referência e desenho de *primer*

Genes de referência candidatos foram selecionados entre os mais comumente usados na literatura. Os *primers* para ciclofilina A (CypA) estavam de acordo com o que foi descrito por Peinnequin et al.^(13)^ e Langnaese et al.^(8)^ Os *primers* de beta-actina (ActB) e ubiquitina C (UbC) foram projetados usando o *software* Primer3, com base em sequências de ratos no banco de dados GenBank. A especificidade dos *primers* foi verificada usando a ferramenta de busca *Basic Local Alignment Search Tool* (BLAST) para coleção de nucleotídeos (nr) do banco de dados do *National Center for Biotechnology Information *(NCBI). Todos os *primers *foram da Invitrogen^®^ (São Paulo, Brasil). A sequência de *primers* usados está descrita na tabela 1.

**Tabela 1:** Genes *housekeeping* selecionados para este estudo, sequências de *primers* e função do gene

HKG	Primer F	Primer R	Função (Langnaese et al.)^(8)^
ActB	5’TATGCCAACACAGTGCTGTCTGG3’	5’TACTCCTGCTTGCTGATCCACAT3’	Proteína do citoesqueleto estrutural
CypA	5’TATCTGCACTGCCAAGACTGAGTG3’	5’CTTCTTGCTGGTCTTGCCATTCC3’	Acelera o dobramento nos oligopeptídeos
UbC	5’TTTCCATAGACAATGCAGATCTTT3’	5’AGGGTGGACTCCTTCTGGAT3’	Degradação de proteínas

ActB: beta-actina; CypA: ciclofilina A; UbC: ubiquitina C.

### Amostras de tecido cerebral

Para determinar a regularidade do ciclo estral, foram colhidos esfregaços vaginais das ratas virgens durante 2 semanas antes do início do experimento. Após a ocorrência de três ciclos estrais regulares, os experimentos foram feitos na manhã da fase diestro. As ratas foram decapitadas e seus cérebros foram rapidamente removidos. As regiões OB, HP, ST e PFC foram dissecadas do hemisfério esquerdo. Logo depois, as amostras dissecadas foram colocadas em tubos contendo RNAlater^®^ (Life Technologies, São Paulo, Brasil) e armazenadas a -80°C.

### Análises moleculares

O RNA total foi extraído de amostras usando TRIzol^®^ (Invitrogen^®^, São Paulo, Brasil). Rapidamente, cada estrutura cerebral foi homogeneizada na presença de TRIzol^®^; acrescentou-se clorofórmio (1:5, v/v) e obteve-se a fase aquosa após centrifugação (12.000×*g*, 15 minutos). O RNA foi precipitado com isopropanol por 15 minutos em temperatura ambiente, seguido por centrifugação a 12.000×*g *por 10 minutos. Os pellets foram novamente suspensos em água tratada com 0,1% dietilpirocarbonato (DEPC) e o RNA foi quantificado por espectrofotometria. A concentração total de RNA foi determinada pela mensuração da densidade óptica a 260nm e a pureza do RNA foi avaliada com base na proporção 260nm/280nm e eletroforese em gel de agarose.^(8)^

A RT-PCR semiquantitativa é bem aceita e amplamente usada como um método valido para análise de expressão gênica nas áreas do cérebro, como já descrito em outros trabalhos.^(14)-16)^ Para a transcrição reversa, 1μg de RNA total foi usado como molde para sintetizar cDNA. RNA foi incubado com 1μL de oligo(dT) (0,5μg/μL, Invitrogen^®^, São Paulo, Brasil), 1μL de desoxirribonucleotídeo trifosfato (dNTPs) (10mM) e água-DEPC para um volume final de 12μL, por 5 minutos a 65°C e, depois, por 1 minuto em gelo. Os seguintes reagentes foram então adicionados para atingir um volume final de 19μL:4μL de tampão RT-PCR (50mM TrisHCl, pH 8,3, 75mM KCl, 3mM MgCl_2_) e 2μL de DTT^®^ (0,1M). Após 2 minutos de incubação a 37°C, 1μL da transcriptase reversa codificada pelo *moloney muri* *ne* *leukemia virus reverse transcriptase* (M-MLV-RT) (200U/μL, Invitrogen^®^, São Paulo, Brasil) foi adicionado e a síntese de cDNA foi feita a 50°C por 1 hora. A reação foi inativada por incubação a 70°C por 15 minutos.

A amplificação dos genes de referência foi feita usando 7,5μL de *SYBR* ^*®*^ *green polymerase chain reaction* *(PCR) master mix* (Applied Biosystems^®^, São Paulo, Brasil), 0,5μL de *primers forward* e *reverse* (0,33µM cada), 100ng de cDNA e água livre de nuclease, em um volume total de 15μL. As reações foram feitas em uma placa óptica de 96 poços, usando um termociclador StepOnePlus^*®*^ (Applied Biosystems^*®*^, Foster City, CA, EUA). Após o passo inicial de desnaturação a 95°C por 10 minutos, a amplificação foi realizada em 50 ciclos de desnaturação a 95°C por 30 segundos, com anelamento de 60°C por 40 segundos e extensão a 72°C por 40 segundos. A amplificação foi seguida por uma análise de curva de fusão para confirmar a especificidade do produto do PCR. Nenhum sinal foi detectado nos controles sem molde *(no-template*). O limiar do ciclo (Ct) experimental foi calculado usando os algoritmos de realce fornecidos pelo equipamento. Todas as amostras foram processadas em duplicata, e o valor médio de cada duplicata foi usado para todos os cálculos restantes.^(8, 10, 17)^

### Determinação da estabilidade de expressão do gene de referência

Foi usada a ferramenta disponível publicamente NormFinder (um aplicativo Visual Basic para Microsoft Excel) para analisar a estabilidade de expressão do gene. Ela faz a classificação dos HKG, segundo sua estabilidade de expressão, usando uma abordagem baseada em modelo. O programa estima a variação de expressão tanto intragrupo como intergrupo e calcula os valores de estabilidade do gene candidato.^(7, 8)^

O relato dos dados obtidos dos valores brutos de Ct representa de maneira falsa a variação e erros, devendo ser evitado. Assim, a expressão de cada HKG foi calculada a partir da fórmula 2^-Ct^. Este cálculo transforma os dados logarítmicos de Ct em um valor linear. A forma 2^-Ct^ descreve, de modo mais preciso, a variação individual entre as reações de replicação.^(6, 18)^ Consequentemente, o NormFinder requer a transformação de valores de Ct em quantidades de expressão numa escala linear, de forma que os valores médios de Ct foram, então, exportados para Microsoft Excel e alterados para a forma 2^-Ct^, e depois as quantidades calculadas foram colocadas no NormFinder,^(19)^ onde o candidato com expressão mais estável no conjunto da amostra é o gene com a primeira colocação, que tem o menor valor de estabilidade.^(20)^

## RESULTADOS

Na classificação de genes candidatos de normalização com o NormFinder, foram obtidos os valores de estabilidade. As figuras 1 a 4 mostram que em HP e ST, o HKG mais estável foi ActB. Por outro lado, Cyp A e UbC foram mais estáveis em PFC e OB, respectivamente.

## DISCUSSÃO

Em alguns estudos de expressão gênica no SNC, ActB se mostrou tão estável quanto gliceraldeído-3-fosfato desidrogenase (GAPDH), mas sua estabilidade depende dos tecidos e das condições usados nas análises.^(21, 22)^ Chen et al.^(23)^ verificaram que outros genes constitutivos, como o fator de alongamento da tradução eucariótica (EF, do inglês *elongation factor*) e GAPDH são mais estáveis do que ActB em algumas áreas do cérebro, como o córtex auditivo e a cóclea de ratos, e que devem, portanto, ser considerados genes de referência em análises quantitativas de expressão gênica nessas regiões. Todavia, muitos estudos baseados em RT-PCR para avaliar a expressão de genes em diferentes regiões do cérebro – como PFC, ST, HP e cerebelo – utilizam ActB como gene de referência.^(14)-16, 24)^

Nossos resultados mostram que ActB é o HKG mais estável no HP do conjunto de nossa amostra. Em um estudo realizado por Langnaese et al.,^(8)^ ActB e CypA provaram ser os genes mais estáveis no HP de sua análise. Esses dois HKG foram também usados como genes de controle em um estudo por Nishida et al.,^(25)^ porque seus níveis de expressão são considerados estáveis nessa área do cérebro. Por outro lado, Honkaniemi et al.^(26)^ usaram apenas CypA em um modelo de isquemia que analisa a expressão gênica no HP.

No ST, também identificamos ActB como o gene mais estável. Contudo, Benn et al.^(9)^ verificaram que UbC é mais estável que ActB nessa área. Honkaniemi et al.^(26)^ examinaram a expressão de CypA para comparar as alterações isquêmicas com os níveis gerais de transcrição no ST.

CypA demonstrou a expressão mais estável no PFC segundo nossa análise. Estudos anteriores de níveis de expressão gênica usaram principalmente um dos HKG mais tradicionais, como GAPDH, 18S ou CypA, no cérebro inteiro de ratos Wistar adultos.^(17)^ Honkaniemi et al.^(26)^ também examinaram a expressão de CypA no córtex de ratos em um modelo isquêmico. Ademais, Benn et al.^(9)^ identificaram UbC como tendo expressão estável no córtex, em vez de ActB. Contudo, Yamada et al.^(27)^ usaram ActB como referência para amplificação de genes, além de Soria-Fregozo et al.,^(14)^ que escolheram ActB como um controle interno para quantificar mRNA no PFC.

Vimos que UbC é o HKG mais estável no OB. UbC já foi usada em vários estudos de expressão gênica, como no cérebro humano^(24, 28)^ e fígado de rato.^(29)^ Todavia, Wong et al.^(30)^ analisaram a expressão de ActB e CypA no OB de ratos.

Nosso estudo teve algumas limitações. Após a extração de RNA com TRIzol^®^, as amostras não foram incubadas com DNAse, mas a pureza do RNA foi verificada por eletroforese em gel de agarose e nenhum DNA estava presente nas amostras. Outra limitação foi o fato de as análises serem baseadas em RT-PCR semiquantitativa. Mesmo que os estudos mais recentes sobre a expressão de genes sejam baseados em PCR quantitativo, muitas investigações foram baseadas em RT-PCR convencional. Aliás, essa foi a técnica molecular para análise de expressão gênica mais usada até recentemente e, portanto, seu uso não prejudica nossos resultados ou outros achados anteriores.

## CONCLUSÃO

As variações na estabilidade dos genes *housekeeping* nas diferentes áreas do sistema nervoso central ocorrem provavelmente devido à variabilidade nas amostras usadas em cada estudo. Assim, a validação dos genes de referência para cada situação experimental é um requisito essencial para aquisição de dados significativos e confiáveis para qualquer tipo de análise. Uma vez selecionado o gene *housekeeping* para cada área do sistema nervoso central, as análises adicionais podem ser feitas com a segurança do uso de um gene de controle mais estável.

**Figura 1:** Estabilidade do gene *housekeeping* no córtex pré-frontal

**Figura 2:** Estabilidade do gene *housekeeping* no hipocampo

**Figura 3:** Estabilidade do gene *housekeeping* no bulbo olfatório

**Figura 4:** Estabilidade do gene *housekeeping* no estriado
